# Heterogeneity of γH2AX Foci Increases in Ex Vivo Biopsies Relative to In Vivo Tumors

**DOI:** 10.3390/ijms19092616

**Published:** 2018-09-04

**Authors:** Treewut Rassamegevanon, Steffen Löck, Michael Baumann, Mechthild Krause, Cläre von Neubeck

**Affiliations:** 1 OncoRay-National Center for Radiation Research in Oncology, Faculty of Medicine and University Hospital Carl Gustav Carus, Technische Universität Dresden, 01307 Dresden, Germany; Treewut.Rassamegevanon@uniklinikum-dresden.de (T.R.); steffen.loeck@oncoray.de (S.L.); Michael.Baumann@dkfz-heidelberg.de (M.B.); Mechthild.Krause@uniklinikum-dresden.de (M.K.); 2 OncoRay-National Center for Radiation Research in Oncology, Helmholtz-Zentrum Dresden-Rossendorf, 01328 Dresden, Germany; 3 Department of Radiotherapy and Radiation Oncology, Faculty of Medicine and University Hospital Carl Gustav Carus, Technische Universität Dresden, 01307 Dresden, Germany; 4 German Cancer Consortium (DKTK), Partner Site Dresden, and German Cancer Research Center (DKFZ), 69120 Heidelberg, Germany; 5 Helmholtz-Zentrum Dresden-Rossendorf, Institute of Radiooncology-OncoRay, 01328 Dresden, Germany; 6 National Center for Tumor Diseases (NCT), Partner Site Dresden, Germany: German Cancer Research Center (DKFZ), 69120 Heidelberg, Germany; 7 National Center for Tumor Diseases (NCT), Partner Site Dresden, Germany: Faculty of Medicine and University Hospital Carl Gustav Carus, Technische Universität Dresden, 01307 Dresden, Germany; 8 National Center for Tumor Diseases (NCT), Partner Site Dresden, Germany: Helmholtz Association/Helmholtz-Zentrum Dresden-Rossendorf (HZDR), 01328 Dresden, Germany; 9 German Cancer Research Center (DKFZ), 69120 Heidelberg, Germany

**Keywords:** radiation, predictive biomarker, DNA damage response, mixed model statistics

## Abstract

The biomarker for DNA double stand breaks, gammaH2AX (γH2AX), holds a high potential as an intrinsic radiosensitivity predictor of tumors in clinical practice. Here, two published γH2AX foci datasets from in and ex vivo exposed human head and neck squamous cell carcinoma (hHNSCC) xenografts were statistically re-evaluated for the effect of the assay setting (in or ex vivo) on cellular geometry and the degree of heterogeneity in γH2AX foci. Significant differences between the nucleus areas of in- and ex vivo exposed samples were found. However, the number of foci increased linearly with nucleus area in irradiated samples of both settings. Moreover, irradiated tumor cells showed changes of nucleus area distributions towards larger areas compared to unexposed samples, implying cell cycle alteration after radiation exposure. The number of residual γH2AX foci showed a higher degree of intra-tumoral heterogeneity in the ex vivo exposed samples relative to the in vivo exposed samples. In the in vivo setting, the highest intra-tumoral heterogeneity was observed in initial γH2AX foci numbers (foci detected 30 min following irradiation). These results suggest that the tumor microenvironment and the culture condition considerably influence cellular adaptation and DNA damage repair.

## 1. Introduction

Cytogenetic biomarkers or molecular (protein-) biomarkers have been extensively investigated in experimental and translational research [1,2,3,4,5] to determine the effect of radiation at the cellular level. However, previous attempts to utilize biomarker data for radiation dose calculation in each individual patient have been unsuccessful. For individualized radiotherapy to be successful, an assay which reliably predicts radiation response is urgently needed to allow for patient stratification and treatment personalization [4,6,7,8,9,10]. GammaH2AX, a phosphorylated variant of the histone H2A family, plays a central role in DNA damage repair pathways. The generation of the phosphorylated histones in the proximity of DNA damage (mainly DNA double strand breaks (DSB)), can be visualized as distinct foci by immunological-based approaches [11]. In addition to the role of γH2AX foci as a DNA DSB biomarker, several biological functions can have an impact on γH2AX foci, e.g., changes in chromosome structure [12] and alteration in the p53-p-21-WIP1 pathway [13]. However, monitoring of γH2AX foci using epifluorescent microscopy is a fast, inexpensive and reliable method for DNA DSB detection [6,8]. Besides the high sensitivity and straightforwardness of the γH2AX foci assay, residual γH2AX foci were demonstrated to correlate with intrinsic radiosensitivity in experimental tumor models [5,14,15], as well as patient-derived materials [16,17,18]. The recently developed ex vivo analysis of γH2AX foci based on tumor biopsies showed promising potential in being used as a clinical predictive assay [5,6,16,17,18]. Despite the high capability of the assay, tumor heterogeneity—one of the major complexities in cancer [19,20,21,22]—challenges the reliability of the assay in radiosensitivity prediction. This leads to controversial concerns addressing the representativeness of ex vivo biopsies relative to in vivo (xenograft) models and patient materials. In addition, the in vivo and ex vivo set-up of the assay relies on γH2AX foci detection in sectioned specimens, leading to only partially analyzed cell nuclei. Thereby, the geometrical changes of cell nuclei due to the in vivo or ex vivo set-up need optimization of γH2AX foci data processing for clinical application of the assay.

This study aims to further optimize the assay by determining the effects of radiation, experimental settings and cellular geometry on γH2AX foci by statistical means. Two published datasets with comparable experimental design but performed with in vivo tumors [14] and ex vivo tumor biopsies [5] were analyzed with a linear mixed-effects model (LMEM) as well as linear regression. The LMEM extends linear regression by including random effect terms in the model. This statistical approach permits the incorporation of multiple sources of variation in the analysis so that complex experimental designs can be comprehensively evaluated and heterogeneity of data can be determined [23,24,25]. Thus, the degree of heterogeneity in γH2AX foci and the influence of the culture condition on the cellular geometry were assessed in two γH2AX foci datasets.

## 2. Results

### 2.1. Impact of Extrinsic Influences on Cellular Geometry

**Figure 1:**
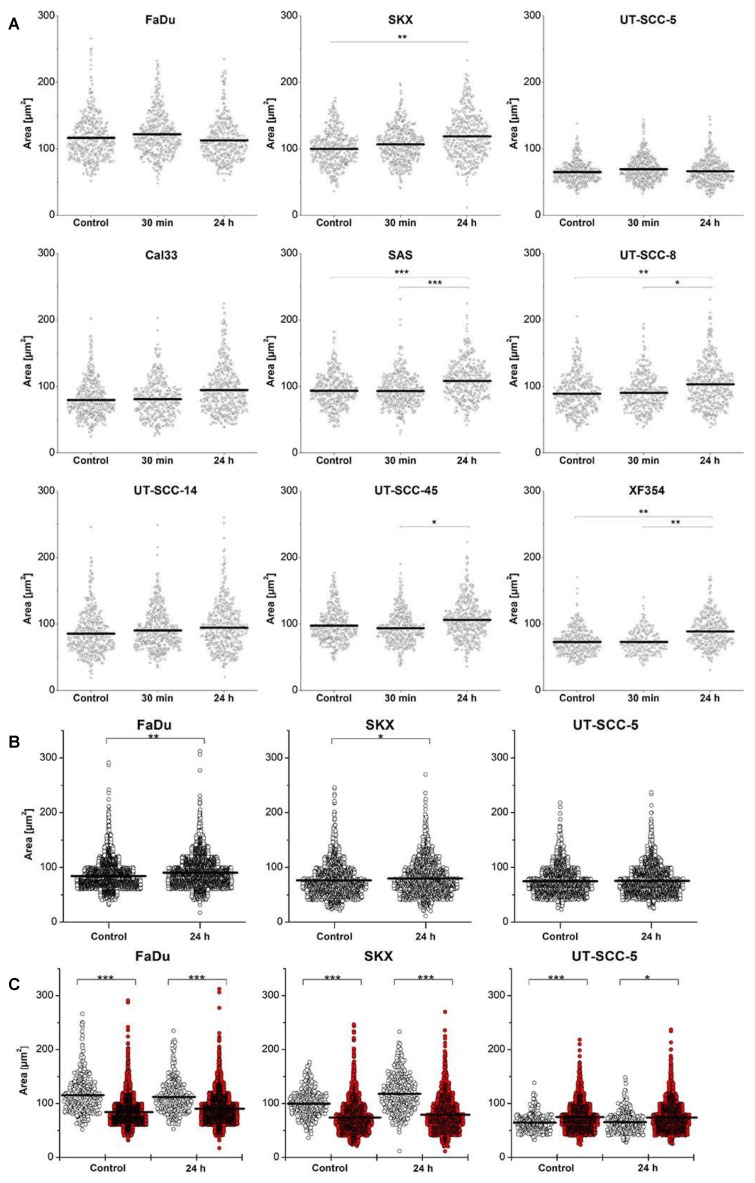
Impact of radiation exposure (4 Gy) and experimental setting (in vivo versus ex vivo) on nucleus area distribution. Scatter plots of nucleus areas from different hHNSCC xenograft models in vivo (**A**) and ex vivo (**B**). Comparison of nucleus areas between the experimental settings (open-circle: in vivo, red: ex vivo) in three hHNSCC tumor lines (FaDu, SKX, UT-SCC-5) (**C**). Nucleus areas were logarithmically transformed and fitted by a linear mixed-effects model (LMEM). Treatment conditions (control, 4 Gy exposure, time point post irradiation) or experimental settings (in vivo versus ex vivo) were defined as fixed effects; tumor, specimen, as well as region of interest (ROI) as random effects. Multiple pairwise comparisons (in vivo) with Bonferroni correction following LMEM analysis were performed. Solid bars represent means. (*: *p* < 0.05, **: *p* < 0.01, ***: *p* < 0.001). *p* values can be found in Supplementary Tables S2 and S3.

In both datasets, cell nucleus areas and foci numbers were determined. The logarithmically transformed nucleus areas of tumors from different hHNSCC models were fitted by the LMEM, where on average (±SD) 415 ± 59 (in vivo) and 3050 ± 1073 (ex vivo) nuclei per tumor model and treatment cohort were analyzed (Table S1). For the in vivo cohort, no statistically significant increase in the mean nucleus area was found in 30 min post exposure samples (4 Gy) relative to unexposed controls. However, the means of nucleus area increased significantly for in vivo (~14%) and ex vivo (~6%) at 24 h post irradiation (4 Gy) in four out of nine (in vivo) and two out of three (ex vivo) evaluated tumor models (Figure 1A,B). The nucleus areas of UT-SCC-5 tumors were not affected in both settings. Independent of treatment, the nucleus areas of FaDu and SKX tumors were statistically significantly larger in the in vivo cohort compared to the ex vivo setting, but smaller in UT-SCC-5 tumors (Figure 1C).

**Figure 2:**
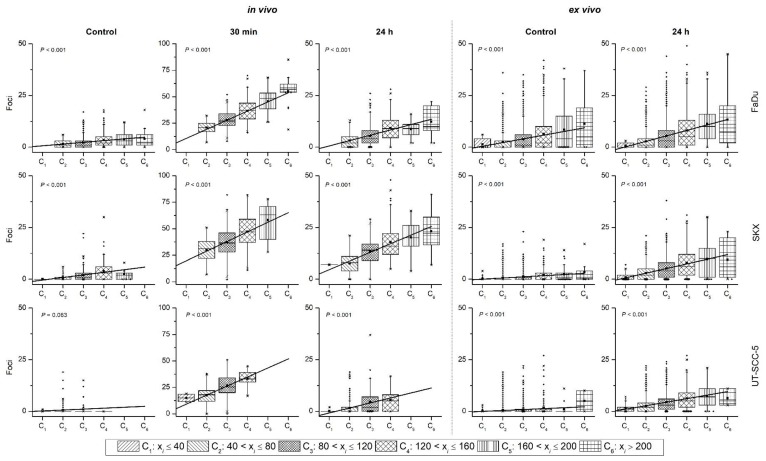
Number of foci linearly increases with the nucleus area (x_i_) categories (C). Exemplary box plots of foci number as a function of the nucleus area categories in FaDu (**upper row**), SKX (**middle row**), and UT-SCC-5 (**lower row**) of control (0 Gy) and exposed (4 Gy) tumors. Nucleus areas were classified into six categories with an interval size of 40 µm^2^. Foci number of controls and exposed tumors, which were fixed 30 min and 24 h post exposure for the in vivo set-up (**left panels**) as well as controls and exposed tumor fixed at 24 h post exposure for the ex vivo set-up (**right panels**) are shown. *P* value of the linear regression analysis is shown. The linear regression analysis outputs as well as data for the remaining six hHNSCC models are presented in supplementary Figure S1 and Table S4. Please note the different Y-axis for 30 min post-exposure in the in vivo set-up.

The nucleus areas were classified in six area categories and foci numbers were plotted accordingly. Linear regression analysis of the nucleus categories and foci numbers showed a significantly linear increase of the foci number with the nucleus area in exposed samples but not in controls for all tumor lines in both cohorts (Figure 2 and Figure S1). To summarize, the experimental setting and the radiation exposure have a significant impact on cell geometry measured as cell nucleus area.

### 2.2. Heterogeneity of γH2AX Foci in Two Experimental Settings

#### 2.2.1. In Vivo Cohort

**Figure 3:**
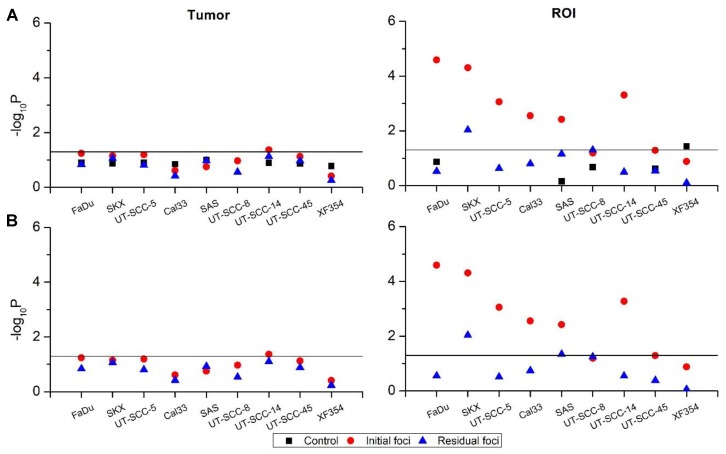
Heterogeneity of corrected foci (cfoci, **upper row**) and normalized foci (nfoci_g_, **lower row**) for the in vivo set-up determined by a linear mixed-effects model (LMEM). Scatter plots of −log_10_*P* values according to the analysis chain of LMEM (black-square: control, red-circle: initial foci, blue-triangle: residual foci) for cfoci (**A**) and nfoci_g_ (**B**). No fixed effect was defined; tumor and ROI were set as random effects. cfoci represents the ratio of foci number and nucleus area of an individual cell multiplied by the mean nucleus area of the corresponding tumor, whereas nfoci is cfoci of an individual nucleus normalized by the mean cfoci of the unexposed tumors (for details see Section 4). Solid lines indicate the significant threshold (*p* = 0.05, above the line: *p* < 0.05). Exact *p* values are given in Supplementary Tables S5 and S6.

The heterogeneity of γH2AX foci was analyzed with LMEM in the in vivo cohort (Figure 3). Foci numbers were corrected and scaled based on the individual nucleus area as well as the mean of the nucleus area of the tumor (cfoci). Normalized foci (nfoci_g_) were calculated by subtracting cfoci of an individual cell with the mean cfoci of the unexposed tumors (for details, see Section 4). The analysis revealed inter-tumoral (among tumors) homogeneity of cfoci in most of the tumor models. This was indicated by insignificant variations in endogenous (control), initial (30 min post exposure) and residual (24 h post exposure) cfoci with the exception of initial cfoci of UT-SCC-14 (Figure 3A, Tumor). In contrast, the variations in cfoci among microscopic regions of interest (ROI) i.e., intra-tumoral heterogeneity were considerably pronounced in initial cfoci (Figure 3A, ROI). However, endogenous and residual cfoci were mostly homogenous among microscopic ROI except endogenous cfoci in XF354 and residual cfoci of SKX. Inter-tumoral homogeneity based on nfoci_g_ was similar to cfoci with initial nfoci_g_ of UT-SCC-14, showing sole significant heterogeneity (Figure 3B, Tumor). Intra-tumoral heterogeneity based on microscopic ROI was found in most of the tumor hHNSCC lines for initial nfoci_g_ and in SKX and SAS tumors for residual nfoci_g_ (Figure 3B, ROI).

#### 2.2.2. Ex Vivo Cohort

**Figure 4:**
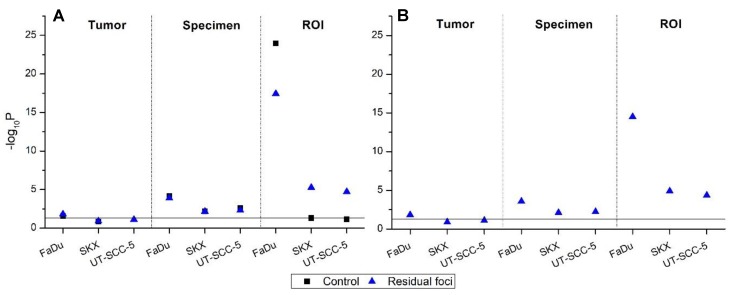
Heterogeneity of corrected foci (cfoci, **A**) and normalized foci (nfoci_g_, **B**) for the ex vivo set-up determined by a linear mixed-effects model (LMEM). Scatter plot of −log_10_*P* value according to the analysis chain of LMEM for cfoci (**A**) and nfoci_g_ (**B**) in three tumor models (black-square: control, blue-triangle: residual foci). No fixed effect was defined; tumor, specimen, and ROI were set as random effects. cfoci represents the ratio of foci number and nucleus area of an individual cell multiplied by mean nucleus area of the corresponding tumor, whereas nfoci is cfoci of individual nuclei normalized by the mean cfoci of unexposed tumors (for details, see Section 4). Solid lines indicate the significant threshold (*p* = 0.05, above the line: *p* < 0.05). Exact *p* values are given in Supplementary Tables S5 and S6.

Inter-tumoral homogeneity of cfoci (among tumors of one hHNSCC model) could be observed in SKX and UT-SCC-5 tumors, but not in FaDu tumors (Figure 4A, Tumor). Intra-tumoral inter-specimen heterogeneity of cfoci (variation among specimens of one tumor) was present in all samples (Figure 4A, Specimen), whereas intra-tumoral intra-specimen heterogeneity of cfoci (variation among ROI of one tumor) was detected in all samples, except endogenous cfoci of SKX and UT-SCC-5 (Figure 4A, ROI). The outcome of the statistical analysis for nfoci_g_ in the ex vivo cohort is similar to the previous report although a different normalization approach was applied [5]. Considering intra-tumoral heterogeneity of cfoci and nfoci_g_ between the in- and ex vivo set-up of the assay, a higher degree of variation can be seen in the ex vivo cohort.

The comparison of cfoci and nfoci_g_ in the in vivo and the ex vivo cohort shows that the results of the γH2AX foci assay are dependent on the experimental setting. An insignificant effect of the culturing condition in residual cfoci and nfoci_g_ was only detected in FaDu tumors (Figure S3). Altogether, these results suggest a higher degree of variation in cfoci and nfoci_g_ of the ex vivo set-up compared to the in vivo set-up of the γH2AX foci assay.

## 3. Discussion

The γH2AX foci assay has been considered as one of the most promising approaches in radiation dosimetry [26,27,28] and intrinsic radiosensitivity prediction [2,3,5,6,14,15,16,17,18,29]. Previously, we showed a significant negative correlation between residual γH2AX foci following in vivo exposure and the TCD_50_ (dose to control 50% of tumors locally) [14]. Furthermore, we demonstrated that the slopes of the dose-response curves of γH2AX foci in ex vivo cultured patient-derived and experimental tumor-derived biopsies differentiated the tumors according to their intrinsic radiosensitivity [16]. The described ex vivo assay relies on ex vivo irradiated tumor specimens of patient or experimental tumors obtained by surgery or punch biopsies. Potential drawbacks for a clinical application of the assay could, however, be the representativeness of a single biopsy and the translatability of ex vivo obtained foci results to an in vivo tumor [30,31]. Here, the representativeness of the γH2AX foci assay was investigated by comparing two published datasets (in vivo [11] and ex vivo [5]), in which the experimental conditions are comparable. In addition, the influence of cellular geometry e.g., the nucleus area, on foci normalization was analyzed. Both questions were statistically approached with a LMEM.

The current study is limited to two experimental datasets generated in a single institution causing an inevitable bias due to e.g., staining procedures, imaging, and γH2AX foci quantification. To the best of our knowledge, there is no published comparable dataset from a different laboratory that is applying the same independent factors i.e., radiation dose, time points, and tumor models. The constant experimental factors minimize their influence on the statistical analysis and enable a unique comparison investigating the effect of the ex vivo cultivation/exposure relative to in vivo exposed tumors. Thus, first conclusions on the influence of the assay setting on the assay readout (γH2AX foci) can be drawn. The results will have major implications: if the ex vivo γH2AX foci assay is not able to reflect the in vivo reaction of the tumor, the assay no longer has the potential to be translated in a clinical routine method.

In a previous publication, γH2AX foci were corrected by the nucleus area and the mean of the nucleus area as weighting and scaling factors, respectively [5,14,16]. Radiation can influence the size of a cell nucleus by e.g., initiating a cell cycle arrest [32,33,34] or alterations in chromatin condensation [35]. Due to the pan-nuclear γH2AX signal in the S-phase of the cell cycle [36,37] and in apoptotic cells [38,39,40], intact- and analyzable nuclei are restricted to G_1_- or G_2_-phase of the cell cycle. In histological sections, a change in nucleus size due to an alteration in the cell cycle would be reflected by an increase or decrease of the nucleus area on average. In clinics, a high variation in tumor cell size, i.e., area, due to inter- and intra-patient heterogeneity should be anticipated. Moreover, the size of the nucleus might change in a dose and time-dependent manner, i.e., intensification and/or protracted release of a G_2_-phase cell cycle arrest after high dose exposure. The incorporation of the nucleus area into a predictive assay or a mathematical model is therefore crucial. Normalizing the foci data on a tumor- and treatment specific mean nucleus area appears to be a reasonable approach for personalized radiosensitivity prediction [5].

Here, we could show alterations of the nucleus area distribution depending on the experimental setting and time after irradiation. In the in vivo and ex vivo cohorts, four out of nine and two out three models showed a significant increase in nucleus area 24 h post exposure (Figure 1), respectively, which was also represented by a switch in cell nucleus categories, i.e., towards higher categories (Figure S2). Both findings most likely indicate a G_2_-phase cell cycle arrest. Interestingly, the change of tumor microenvironment (from in vivo to ex vivo) induced a significant reduction in cell nucleus area in FaDu and SKX, while UT-SCC-5 tumors increased their cell nucleus areas (Figure 1C) independent of the radiation treatment. Especially, the area changes seen in control samples suggest that cells adapted to the new culture condition by modulating proliferation and progression through the cell cycle.

Determination of intrinsic radiosensitivity by γH2AX cfoci and nfoci on tumor sections relies on immune-histopathological approaches [5,14,16,17]. Relative to the in vitro situation where foci formation of an entire nucleus can be observed, solid tissue sections have a common limitation: the representation of a three-dimensional tumor structure in a two-dimensional top-view projection. Cells and cell nuclei are sectioned in different anatomical planes depending on the positioning of tissue during the embedding process. This circumstance is more relevant in tumors, as nucleus size variation is a common feature [41,42,43]. This study demonstrated a linear correlation between foci number and nucleus area after DNA damage induction, indicating a dependency of foci detection on measured nucleus area. This emphasizes the relevance of incorporation of a cellular geometry in the data processing chain [44] by applying e.g., a LMEM. Analysis of foci cluster, size, or volume could potentially provide additional information on (complex-) DNA damage and intrinsic radiosensitivity, since foci are significantly dependent on radiation quality [44,45] and cell lines [46]. This indicates a functionality of foci geometry as an indicator for DNA damage complexities [47]. Estimating foci volume in conjunction with foci number per nucleus area in tumor specimens could, therefore, be a promising approach in the assessment of tumor radiation response.

Following ionizing radiation, the γH2AX foci signal reaches the intensity peak at approximately 30 min post DNA damage induction in a dose-dependent manner [48,49]. Pronounced intra-tumoral heterogeneity of initial cfoci and nfoci could be seen in the in vivo cohort for most of the evaluated tumor models (Figure 3A,B). The indistinctive morphology of foci at early time points post exposure i.e., overlapping foci and giant foci, contributes to the data variation [14,33,48], which might call for a higher sample size for initial foci detection to minimize the variability.

Tumor heterogeneity is one of the common complexities in cancer research and therapy [19,20]. Generally, a higher degree of heterogeneity would be expected in vivo, where a plethora of uncontrollable factors e.g., oxygen supply, nutrition supply, and tumor microenvironment can potentially affect the biological read-out [30,31,50,51]. However, in this study, both cfoci and nfoci_g_ showed a more pronounced heterogeneity in the ex vivo set-up, compared to the in vivo set-up. Based on these results, dissecting and transferring a tumor sample into a new microenvironment with supposable optimal oxygen and nutrition supply led to the intensification of heterogeneity in γH2AX foci. An asynchronous adaptation of cells to the ex vivo culture condition could possibly contribute to the higher degree of heterogeneity in γH2AX foci. A LMEM approach includes random and fixed effects of a data acquisition chain, which are frequently disregarded in classical parametric or non-parametric models, although they might have an influence on the statistical outcome [5,23,24,25]. In this study, main contributors for data variation were intra-tumoral (in vivo), and intra-tumoral/inter- and intra specimen (ex vivo). As a consequence, certain amounts of tumor specimen, ROI, and nuclei should be analyzed to obtain a precise prediction of the radiation response measured with γH2AX foci [5,17]. Indications for an adjustment of the foci analysis from the in vivo to the ex vivo situation can be drawn from the FaDu, SKX and UT-SCC-5 cohorts. For the latter two models, a significant difference in residual nfoci_g_ was found, while for FaDu no significant difference in residual nfoci_g_ between the in vivo and ex vivo set-up was detected (Figure S3). Considering the sample size of FaDu (Table S1), for the ex vivo cohort ~ ten times more nuclei were analyzed for foci relative to the in vivo cohort (4390 versus 450 nuclei); for SXK and UT-SCC-5 ~ five to seven times more nuclei were scored for foci (2000 versus 450 nuclei (SKX), 2830 versus 400 nuclei (UT-SCC-5)). This implies that a sample size of ≥ 10 for the ex vivo situation is needed to obtain comparable results to the in vivo situation.

In summary, this statistical study applied a LMEM to show that the nucleus area and the heterogeneity of γH2AX foci in hHNSCC models depends on the experimental set-up (in vivo, ex vivo) and possibly on the cellular adaptation of the tumor specimen to the new microenvironment [50,52]. From the translational perspective, patient-derived materials are expected to have a higher degree of heterogeneity compared to preclinical in vivo models, where clonal selection during passaging potentially diminishes the native tumor heterogeneity [21,31,50,51,52,53]. Thereby, multiple samples, ROI per specimen, and nuclei per ROI should be accounted for in γH2AX foci analysis [5]. For a precise and non-biased radiosensitivity prediction, a sophisticated semi-automated foci counting software is necessary for bridging the gap between laboratory and clinic.

## 4. Materials and Methods

### 4.1. Tumor Handling and Data Acquisition

**Figure 5:**
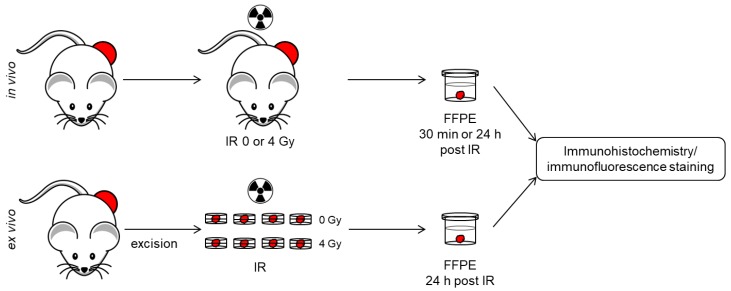
Overview of experimental designs applied in the published datasets of the in vivo [14] and ex vivo (5] cohort. For the in vivo set-up, tumor-bearing mice were exposed to X-ray irradiation (IR). Tumors were excised, fixed in formalin, and embedded in paraffin (FFPE) 30 min or 24 h post IR (**upper panel**). For the ex vivo set-up, tumor specimen were excised, cultured in petri-dished containing medium, and irradiated. Specimens were fixed and embedded 24 h post IR (**lower panel**). Immunohistochemistry and immunofluorescence staining were performed with an identical procedure. Foci evaluation was performed exclusively in oxic and viable parts of the tumor.

The heterogeneity of γH2AX foci from in vivo irradiated tumors [14] and ex vivo irradiated tumor biopsies [5] were assessed in two published datasets. In both studies, hHNSCC tumor xenografts were used. GammaH2AX foci analysis was solely performed in oxic and viable tumor regions based on immunohistochemical detection of pimonidazole and bromodeoxyuridine (BrdU) incorporation. Nucleus area was extracted from the DNA staining with DAPI (Figure 5, graphical abstract).

The in vivo cohort comprising nine hHNSCC tumor xenograft models (Cal33, FaDu, SAS, SKX, XF354, UT-SCC-5, UT-SCC-8, UT-SCC-14, UT-SCC-45) were exposed to 4 Gy, excised and fixed 30 min or 24 h post exposure. GammaH2AX foci were analyzed in 4–10 tumors/model and in 5 randomly selected nuclei from 10 oxic ROI/tumor (50 nuclei/tumor) [14]. For the ex vivo cohort, up to sixteen tumor specimens from subcutaneous hHNSCC tumors (SKX, FaDu, UT-SCC-5) were taken and cultured ex vivo. Half of the specimens/tumor were exposed to 4 Gy. All samples were fixed and paraffin embedded 24 h later. GammaH2AX foci were scored in 10 randomly selected nuclei in 5–7 oxic ROI/specimen (50–70 nuclei/specimen) [5].

### 4.2. Data Processing

Foci number from both datasets were corrected and normalized as described previously [5]. In brief, corrected foci were calculated as:

(1) $$\mathrm{cfoci}=\frac{{\mathrm{foci}}_{i}}{{\mathrm{area}}_{i}}\times \bar{{\mathrm{area}}_{t}}$$

where ${\mathrm{area}}_{i}$ is the area of an individual cell nucleus and the corresponding foci number ${\mathrm{foci}}_{i}$. $\bar{{\mathrm{area}}_{t}}$ is the tumor- and dose specific mean nucleus area of the corresponding tumor. Due to the experimental setting, a tumor-specific unexposed control is unavailable for the in vivo set-up. Therefore, the overall mean of the corrected foci of all control tumors (cfoci_0_) was calculated for each tumor model and used for normalizing the corrected foci of a radiation exposed sample as follows:

(2) $${\mathrm{nfoci}}_{g}=\mathrm{cfoci}-\bar{{\mathrm{cfoci}}_{0}}$$

In the following, negative nfoci_g_ were set to zero under the assumption that radiation does not induce negative DNA damage. Statistical analysis was performed with SPSS 24 (IBM Deutschland GmbH, Ehningen, Germany). Graphs were plotted with OriginPro 8G (OriginLab Corp. Northampton, MA, USA).

### 4.3. Statistical Analysis

#### 4.3.1. Analysis on Cellular Geometry and Foci Number

Alterations in the nucleus area are crucial in the correction and normalization procedures of γH2AX foci data. Therefore, extrinsic influences (i.e., radiation dose, experimental cohort) on the nucleus area were analyzed by fitting the log-transformed nucleus area by the LMEM. The nucleus area distribution was determined by classifying the data into six categories with a bin size of 40 µm^2^. The foci number of individual nuclei was grouped according to the categories. Linear regression analysis was performed to describe the correlation between foci number and nucleus area categories.

#### 4.3.2. Heterogeneity of γH2AX Foci

The LMEM was applied to determine inter-tumoral (between tumors of a model) and intra-tumoral (within one tumor) heterogeneity. Fixation time points (control, 30 min (only in vivo), 24 h) or experimental settings (in vivo, ex vivo) were defined as fixed effects, whereas tumor, specimen (only ex vivo), and ROI were defined as random effects. Since the in vivo cohort only comprises of tumor and ROI but not a specimen in the analysis chain, ROI and tumor were repeatedly included to generate the missing factor in the LMEM for direct comparison between the experimental settings. In case of data fitting without a fixed effect, restricted maximum likelihood estimation (REML) was applied. Otherwise, maximum likelihood estimation (ML) was employed. For multiple pairwise comparisons, Bonferroni adjustment was conducted. The varying reoxygenation times in the ex vivo cohort were excluded from the analysis, as an insignificant influence was previously found [5]. The statistically significant threshold was defined as *p* ≤ 0.05. The *p* values of the statistical analyses and input datasets are presented in supplementary information (Tables S1, S5–S7).

## Supplementary Materials

Supplementary materials can be found at http://www.mdpi.com/1422-0067/19/9/2616/s1.

## Abbreviations

γH2AX	Gamma H2AX
hHNSCC	Human head and neck squamous cell carcinoma
LMEM	Linear mixed effects model
ROI	Region of interest
REML	Restricted maximum likelihood estimation
ML	Maximum likelihood estimation
BrdU	Bromodeoxyuridine
cfoci	Corrected foci
nfoci	Normalized foci
