# Supplementary material for: Heterogeneity of γH2AX Foci Increases in Ex Vivo Biopsies Relative to In Vivo Tumors
Source: Int J Mol Sci. 2018 Sep 4;19(9):2616. doi: 10.3390/ijms19092616 (PMC6163410; doi:10.3390/ijms19092616)
Supplement: Supplementary file 1 [file ijms-19-02616-s001.pdf]

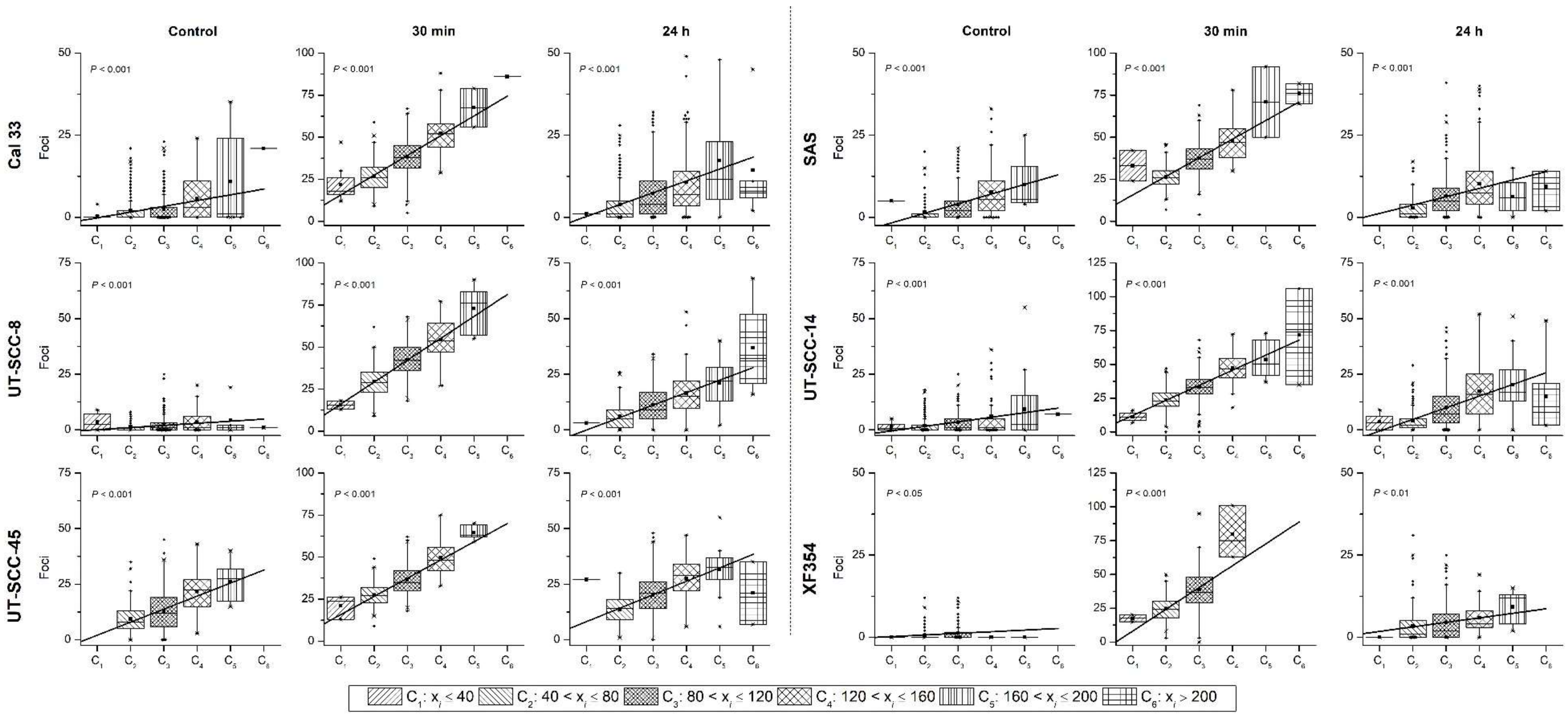


**Figure S1** Number of foci linearly increase with the nucleus area ($x_i$) categories (C). Box plots of foci number of six hHNSCC models were classified into six nucleus area categories with an interval size of 40 µm². Foci number of controls and exposed (4 Gy) tumors, which were fixed 30 min and 24 h post exposure for the *in vivo* set-up are shown. *P* value of linear regression analysis is shown. Linear regression analysis outputs is presented in supplementary Table S4. Please note the different Y-axis for 30 min post-exposure.

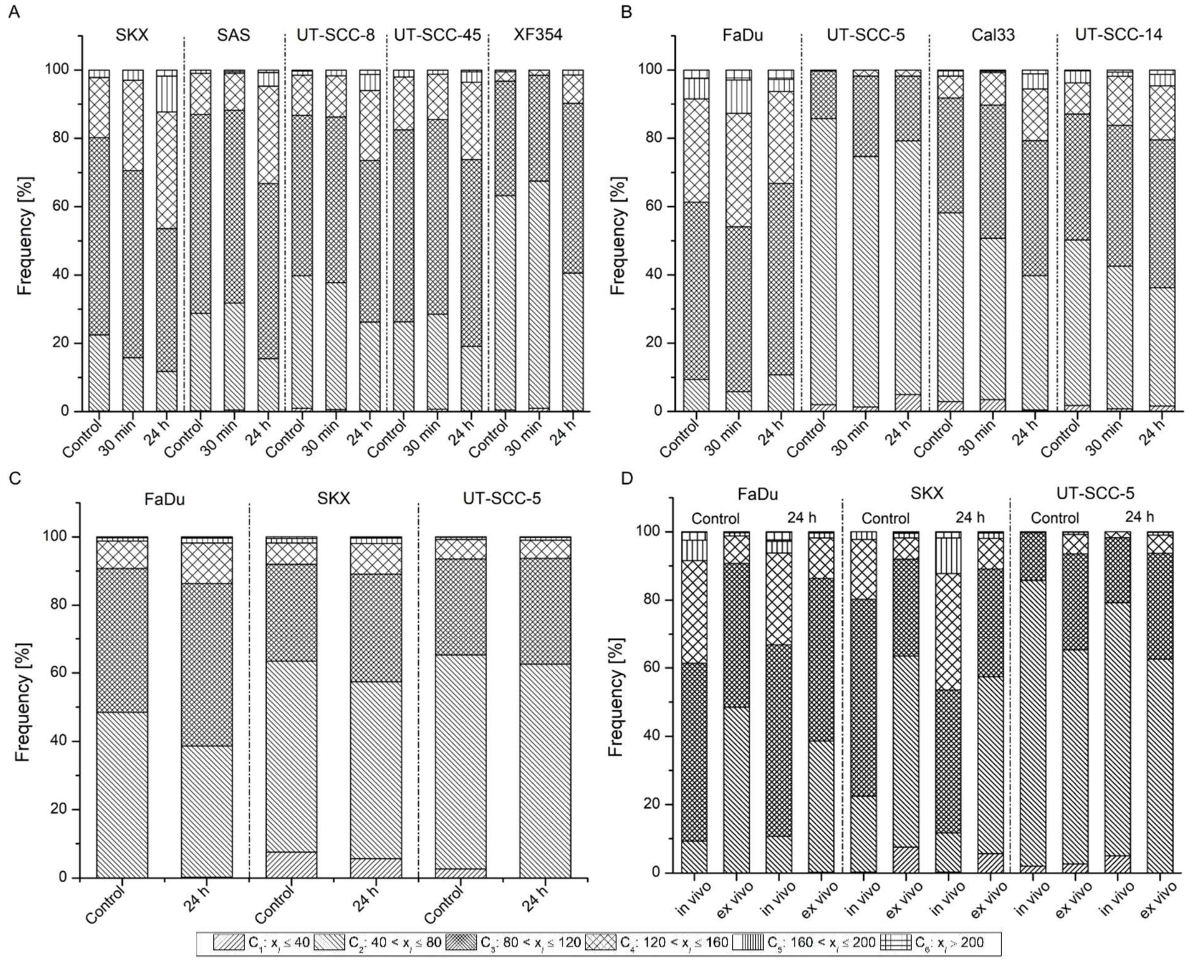


**Figure S2** Culmulative frequency percentage plots of nucleus area ($x_i$) classified into six categories with a bin size of 40 $\mu m^2$. Nucleus area distribution for the *in vivo* set-up of which the statistical anlaysis by LMEM showed significant (A) and insignificant (B) differences among treatment groups, and for the *ex vivo* set-up (C). Comparison of the culmulative percentage nucleus area distribution between the experimental settings in three tumor lines (D).

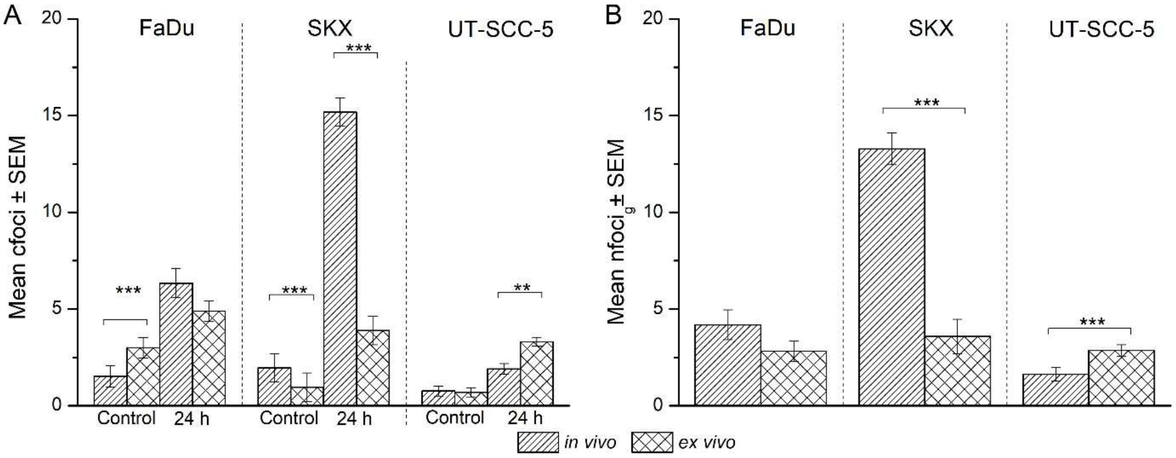


**Figure S3**. Comparison of mean cfoci ± standard error of mean (SEM) (A) and nfoci$_g$ ± SEM (B) in three tumor models subjected to both experimental settings of the γH2AX assay. Data were fitted into LMEM where the experimental setting was defined as fixed effect and tumor, specimen, and ROI were defined as random effects. (**: $P < 0.01$, ***: $P < 0.001$). ROI was repeatedly included to generate the missing factor (specimen) for the *in vivo* set-up. Exact $P$ values are presented in Supplementary Table S7.

**Table S1**. Summary of the input data for the statistical analysis

| | Tumor line | Condition | No. of tumors | No. of specimen | No. of ROI | Total data input |
|---|---|---|---|---|---|---|
| *in vivo* | FaDu | Control | 9 | - | 90 | 450 |
| | | 30 min post IR | 9 | - | 90 | 450 |
| | | 24 h post IR | 8 | - | 80 | 400 |
| | SKX | Control | 9 | - | 90 | 450 |
| | | 30 min post IR | 10 | - | 100 | 500 |
| | | 24 h post IR | 9 | - | 90 | 450 |
| | UT-SCC-5 | Control | 8 | - | 80 | 400 |
| | | 30 min post IR | 9 | - | 90 | 450 |
| | | 24 h post IR | 8 | - | 80 | 400 |
| | Cal33 | Control | 9 | - | 90 | 450 |
| | | 30 min post IR | 8 | - | 80 | 400 |
| | | 24 h post IR | 9 | - | 90 | 450 |
| | SAS | Control | 8 | - | 80 | 400 |
| | | 30 min post IR | 8 | - | 80 | 400 |
| | | 24 h post IR | 8 | - | 80 | 400 |
| | UT-SCC-8 | Control | 8 | - | 80 | 400 |
| | | 30 min post IR | 7 | - | 70 | 350 |
| | | 24 h post IR | 9 | - | 90 | 450 |
| | UT-SCC-14 | Control | 9 | - | 90 | 450 |
| | | 30 min post IR | 10 | - | 100 | 500 |
| | | 24 h post IR | 9 | - | 90 | 450 |
| | UT-SCC-45 | Control | 8 | - | 80 | 400 |
| | | 30 min post IR | 8 | - | 80 | 400 |
| | | 24 h post IR | 9 | - | 90 | 450 |
| | XF354 | Control | 7 | - | 70 | 350 |
| | | 30 min post IR | 4 | - | 40 | 200 |
| | | 24 h post IR | 7 | - | 70 | 350 |
| *ex vivo* | FaDu | Control | 16 | 60 | 438 | 4380 |
| | | 24 h post IR | 16 | 60 | 439 | 4390 |
| | SKX | Control | 9 | 31 | 217 | 2170 |
| | | 24 h post IR | 9 | 31 | 200 | 2000 |
| | UT-SCC-5 | Control | 10 | 39 | 253 | 2530 |
| | | 24 h post IR | 10 | 40 | 283 | 2830 |

IR: Irradiation

**Table S2.** Summary of the statistical output of nucleus area analyzed by a LMEM. Bonferroni correction was applied for multiple comparisons.

| | Tumor line | Condition | Mean | Standard deviation | *P* value[a] | | |
|---|---|---|---|---|---|---|---|
| | | | | | Control | 30 min | 24 h |
| *in vivo* | FaDu | Control | 116.4466 | 33.34720 | - | 0.804 | 1.000 |
| | | 30 min post IR | 122.2892 | 32.38627 | - | - | 0.297 |
| | | 24 h post IR | 112.6412 | 30.59298 | - | - | - |
| | SKX | Control | 100.2680 | 25.86748 | - | 0.281 | 0.001 |
| | | 30 min post IR | 107.1704 | 26.17204 | - | - | 0.084 |
| | | 24 h post IR | 119.1873 | 33.93800 | - | - | - |
| | UT-SCC-5 | Control | 65.2466 | 15.69384 | - | 0.701 | 1.000 |
| | | 30 min post IR | 69.4444 | 18.16860 | - | - | 0.739 |
| | | 24 h post IR | 66.1853 | 19.15192 | - | - | - |
| | Cal33 | Control | 80.2138 | 27.38123 | - | 1.000 | 0.120 |
| | | 30 min post IR | 81.1026 | 28.31106 | - | - | 0.156 |
| | | 24 h post IR | 94.9764 | 35.76368 | - | - | - |
| | SAS | Control | 93.8444 | 24.26456 | - | 1.000 | 0.000 |
| | | 30 min post IR | 93.5702 | 24.84296 | - | - | 0.000 |
| | | 24 h post IR | 108.7612 | 28.34736 | - | - | - |
| | UT-SCC-8 | Control | 89.5806 | 28.27604 | - | 1.000 | 0.004 |
| | | 30 min post IR | 90.7311 | 26.55216 | - | - | 0.017 |
| | | 24 h post IR | 103.3802 | 33.47841 | - | - | - |
| | UT-SCC-14 | Control | 85.7874 | 31.78737 | - | 1.000 | 0.787 |
| | | 30 min post IR | 90.5895 | 29.16455 | - | - | 1.000 |
| | | 24 h post IR | 94.9591 | 35.63261 | - | - | - |
| | UT-SCC-45 | Control | 97.3420 | 24.88855 | - | 1.000 | 0.109 |
| | | 30 min post IR | 93.9249 | 23.99820 | - | - | 0.011 |
| | | 24 h post IR | 106.2284 | 28.73631 | - | - | - |
| | XF354 | Control | 73.1351 | 19.25533 | - | 1.000 | 0.001 |
| | | 30 min post IR | 73.2460 | 17.68541 | - | - | 0.004 |
| | | 24 h post IR | 89.1600 | 23.93514 | - | - | - |
| *ex vivo* | FaDu | Control | 85.596336 | 25.218263 | - | - | 0.001 |
| | | 24 h post IR | 91.355702 | 26.967857 | - | - | - |
| | SKX | Control | 75.658134 | 30.057774 | - | - | 0.040 |
| | | 24 h post IR | 79.744765 | 31.672199 | - | - | - |
| | UT-SCC-5 | Control | 75.021162 | 25.737746 | - | - | 0.522 |
| | | 24 h post IR | 75.603470 | 26.228616 | - | - | - |

a Statistical significance was determined by fitting logarithmic nucleus area into a LMEM with condition as fixed effect and tumor and ROI as random effects. IR: Irradiation

**Table S3.** Statistical output of the comparison between the experimental settings of logarithmic nucleus area in three tumor lines. Tumor or ROI was assigned as random effects in the LMEM repeatedly to complete the missing factor for *in vivo* set-up.

| | Tumor line | Condition | Experimental set-up (I) | Experimental set-up (J) | Mean Difference (I-J) | Standard error of mean | Degree of Freedom | *P* Value |
|---|---|---|---|---|---|---|---|---|
| Repeat tumor | FaDu | Control | *in vivo* | *ex vivo* | 0.132 | 0.008 | 2536.809 | 0.000 |
| | | 24 h | *in vivo* | *ex vivo* | 0.095 | 0.018 | 108.437 | 0.000 |
| | SKX | Control | *in vivo* | *ex vivo* | 0.137 | 0.011 | 738.752 | 0.000 |
| | | 24 h | *in vivo* | *ex vivo* | 0.186 | 0.019 | 44.917 | 0.000 |
| | UT-SCC-5 | Control | *in vivo* | *ex vivo* | -0.006 | 0.009 | 1134.012 | 0.515 |
| | | 24 h | *in vivo* | *ex vivo* | -0.076 | 0.028 | 35.043 | 0.011 |
| Repeat ROI | FaDu | Control | *in vivo* | *ex vivo* | 0.123 | 0.007 | 2241.822 | 0.000 |
| | | 24 h | *in vivo* | *ex vivo* | 0.092 | 0.012 | 41.231 | 0.000 |
| | SKX | Control | *in vivo* | *ex vivo* | 0.141 | 0.010 | 1121.132 | 0.000 |
| | | 24 h | *in vivo* | *ex vivo* | 0.187 | 0.017 | 20.967 | 0.000 |
| | UT-SCC-5 | Control | *in vivo* | *ex vivo* | -0.043 | 0.008 | 1502.768 | 0.000 |
| | | 24 h | *in vivo* | *ex vivo* | -0.054 | 0.024 | 19.827 | 0.033 |

**Table S4.** Linear regression coefficient and $R^2$ of $\gamma$H2AX foci number as a function of nucleus area categories from different tumor lines and treatment conditions

| | Tumor line | Condition | Slope | Sig. (Slope) | Intercept | Sig. (Intercept) | R Square |
|---|---|---|---|---|---|---|---|
| *in vivo* | FaDu | Control | 0.836 | 0.000 | -0.231 | 0.716 | 0.046 |
| | | 30 min post IR | 8.624 | 0.000 | 2.229 | 0.240 | 0.383 |
| | | 24 h post IR | 2.582 | 0.000 | -2.038 | 0.062 | 0.141 |
| | SKX | Control | 1.256 | 0.000 | -1.710 | 0.007 | 0.077 |
| | | 30 min post IR | 9.090 | 0.000 | 10.581 | 0.000 | 0.193 |
| | | 24 h post IR | 4.161 | 0.000 | 0.610 | 0.641 | 0.227 |
| | UT-SCC-5 | Control | 0.447 | 0.063 | -0.323 | 0.533 | 0.009 |
| | | 30 min post IR | 8.560 | 0.000 | 0.546 | 0.769 | 0.202 |
| | | 24 h post IR | 2.423 | 0.000 | -3.169 | 0.000 | 0.097 |
| | Cal33 | Control | 1.750 | 0.000 | -1.782 | 0.026 | 0.068 |
| | | 30 min post IR | 11.749 | 0.000 | 3.815 | 0.038 | 0.424 |
| | | 24 h post IR | 3.616 | 0.000 | -3.436 | 0.010 | 0.131 |
| | SAS | Control | 2.924 | 0.000 | -4.669 | 0.000 | 0.127 |
| | | 30 min post IR | 11.078 | 0.000 | 4.342 | 0.027 | 0.402 |
| | | 24 h post IR | 2.548 | 0.000 | -1.263 | 0.395 | 0.076 |
| | UT-SCC-8 | Control | 0.954 | 0.000 | -0.739 | 0.241 | 0.044 |
| | | 30 min post IR | 13.023 | 0.000 | 3.265 | 0.117 | 0.481 |
| | | 24 h post IR | 5.601 | 0.000 | -5.535 | 0.000 | 0.264 |
| | UT-SCC-14 | Control | 2.046 | 0.000 | -2.374 | 0.006 | 0.090 |
| | | 30 min post IR | 11.170 | 0.000 | 0.774 | 0.638 | 0.432 |
| | | 24 h post IR | 5.242 | 0.000 | -5.701 | 0.000 | 0.214 |
| | UT-SCC-45 | Control | 5.855 | 0.000 | -3.560 | 0.045 | 0.200 |
| | | 30 min post IR | 10.828 | 0.000 | 4.836 | 0.007 | 0.442 |
| | | 24 h post IR | 6.041 | 0.000 | 2.127 | 0.214 | 0.222 |
| | XF354 | Control | 0.498 | 0.010 | -0.421 | 0.360 | 0.019 |
| | | 30 min post IR | 16.135 | 0.000 | -7.835 | 0.051 | 0.320 |
| | | 24 h post IR | 1.376 | 0.001 | 0.487 | 0.665 | 0.032 |
| *ex vivo* | FaDu | Control | 1.792 | 0.000 | -1.289 | 0.000 | 0.051 |
| | | 24 h post IR | 2.652 | 0.000 | -2.570 | 0.000 | 0.100 |
| | SKX | Control | 0.562 | 0.000 | -0.415 | 0.005 | 0.041 |
| | | 24 h post IR | 2.239 | 0.000 | -1.448 | 0.000 | 0.136 |
| | UT-SCC-5 | Control | 0.445 | 0.000 | -0.373 | 0.028 | 0.016 |
| | | 24 h post IR | 1.706 | 0.000 | -0.743 | 0.003 | 0.091 |

IR: Irradiation

**Table S5.** Summary of the statistical analysis output analyzed by a random effect model for cfoci from the *in vivo* and *ex vivo* datasets

| | Tumor line | Condition | Mean | Standard error of mean | P value | | | |
|---|---|---|---|---|---|---|---|---|
| | | | | | Tumor | Tumor x ROI | Tumor x Specimen | Tumor x Specimen x ROI |
| *in vivo* | FaDu | Control | 2.569 | 1.335 | 0.128 | 0.136 | - | - |
| | | 30 min post IR | 33.216 | 1.335 | 0.057 | 0.000 | - | - |
| | | 24 h post IR | 6.335 | 1.416 | 0.144 | 0.300 | - | - |
| | SKX | Control | 1.939 | 1.303 | 0.134 | [a] | - | - |
| | | 30 min post IR | 39.707 | 1.236 | 0.070 | 0.000 | - | - |
| | | 24 h post IR | 15.186 | 1.303 | 0.086 | 0.009 | - | - |
| | UT-SCC-5 | Control | 0.599 | 1.013 | 0.127 | [a] | - | - |
| | | 30 min post IR | 19.984 | 0.955 | 0.064 | 0.001 | - | - |
| | | 24 h post IR | 1.908 | 1.013 | 0.155 | 0.234 | - | - |
| | Cal33 | Control | 2.473 | 0.663 | 0.142 | [a] | - | - |
| | | 30 min post IR | 34.516 | 0.703 | 0.245 | 0.003 | - | - |
| | | 24 h post IR | 6.596 | 0.663 | 0.382 | 0.160 | - | - |
| | SAS | Control | 3.404 | 0.848 | 0.101 | 0.695 | - | - |
| | | 30 min post IR | 35.748 | 0.848 | 0.178 | 0.004 | - | - |
| | | 24 h post IR | 6.417 | 0.848 | 0.108 | 0.070 | - | - |
| | UT-SCC-8 | Control | 1.880 | 1.103 | [a] | 0.213 | - | - |
| | | 30 min post IR | 39.868 | 1.180 | 0.107 | 0.064 | - | - |
| | | 24 h post IR | 11.156 | 1.040 | 0.276 | 0.050 | - | - |
| | UT-SCC-14 | Control | 2.847 | 1.568 | 0.128 | [a] | - | - |
| | | 30 min post IR | 31.766 | 1.488 | 0.043 | 0.000 | - | - |
| | | 24 h post IR | 8.956 | 1.568 | 0.074 | 0.320 | - | - |
| | UT-SCC-45 | Control | 13.367 | 1.231 | 0.137 | 0.237 | - | - |
| | | 30 min post IR | 36.032 | 1.231 | 0.074 | 0.051 | - | - |
| | | 24 h post IR | 21.072 | 1.160 | 0.108 | 0.290 | - | - |
| | XF354 | Control | 0.723 | 0.576 | 0.166 | 0.037 | - | - |
| | | 30 min post IR | 29.431 | 0.762 | 0.384 | 0.131 | - | - |
| | | 24 h post IR | 4.117 | 0.576 | 0.559 | 0.792 | - | - |
| *ex vivo* | FaDu | Control | 3.343 | 0.672 | 0.028 | - | 0.000 | 0.000 |
| | | 24 h post IR | 4.715 | 0.817 | 0.015 | - | 0.000 | 0.000 |
| | SKX | Control | 0.931 | 0.216 | 0.146 | - | 0.007 | 0.050 |
| | | 24 h post IR | 4.208 | 0.680 | 0.123 | - | 0.007 | 0.000 |
| | UT-SCC-5 | Control | 0.666 | 0.084 | [a] | - | 0.003 | 0.070 |
| | | 24 h post IR | 3.348 | 0.353 | 0.079 | - | 0.005 | 0.000 |

a: This covariance parameter is redundant. The test statistic and confidence interval cannot be computed. IR: Irradiation

**Table S6.** Summary of the statistical analysis output analyzed by a random effect model for $nfoci_g$ from the *in vivo* and *ex vivo* datasets

| | Tumor line | Condition | Mean | Standard error of mean | *P* value | | | |
|---|---|---|---|---|---|---|---|---|
| | | | | | Tumor | Tumor x ROI | Tumor x Specimen | Tumor x Specimen x ROI |
| *in vivo* | FaDu | 30 min post IR | 30.648 | 2.330 | 0.057 | 0.000 | - | - |
| | | 24 h post IR | 4.191 | 0.521 | 0.144 | 0.284 | - | - |
| | SKX | 30 min post IR | 37.768 | 1.944 | 0.070 | 0.000 | - | - |
| | | 24 h post IR | 13.286 | 1.060 | 0.087 | 0.009 | - | - |
| | UT-SCC-5 | 30 min post IR | 19.391 | 1.642 | 0.064 | 0.001 | - | - |
| | | 24 h post IR | 1.623 | 0.361 | 0.156 | 0.308 | - | - |
| | Cal33 | 30 min post IR | 32.043 | 1.093 | 0.245 | 0.003 | - | - |
| | | 24 h post IR | 4.839 | 0.527 | 0.384 | 0.181 | - | - |
| | SAS | 30 min post IR | 32.344 | 1.092 | 0.178 | 0.004 | - | - |
| | | 24 h post IR | 3.800 | 0.778 | 0.118 | 0.045 | - | - |
| | UT-SCC-8 | 30 min post IR | 37.988 | 2.199 | 0.107 | 0.064 | - | - |
| | | 24 h post IR | 9.411 | 0.607 | 0.292 | 0.056 | - | - |
| | UT-SCC-14 | 30 min post IR | 28.750 | 2.332 | 0.043 | 0.001 | - | - |
| | | 24 h post IR | 6.547 | 1.124 | 0.078 | 0.284 | - | - |
| | UT-SCC-45 | 30 min post IR | 22.665 | 1.791 | 0.074 | 0.051 | - | - |
| | | 24 h post IR | 8.619 | 0.773 | 0.130 | 0.410 | - | - |
| | XF354 | 30 min post IR | 28.711 | 1.669 | 0.384 | 0.132 | - | - |
| | | 24 h post IR | 3.611 | 0.341 | 0.581 | 0.872 | - | - |
| *ex vivo* | FaDu | 24 h post IR | 2.823 | 0.642 | 0.014 | - | 0.000 | 0.000 |
| | SKX | 24 h post IR | 3.598 | 0.625 | 0.126 | - | 0.007 | 0.000 |
| | UT-SCC-5 | 24 h post IR | 2.862 | 0.329 | 0.079 | - | 0.006 | 0.000 |

IR: Irradiation

**Table S7A**. Summary of the statistical analysis output analyzed by LMEM for cfoci for direct comparison between *in vivo* and *ex vivo* datasets. Tumor or ROI was assigned as random effects in the LMEM repeatedly to complete the missing factor for *in vivo* set-up

| | Tumor line | Condition | Experimental set-up (I) | Experimental set-up (J) | Mean Difference (I-J) | Standard error of mean | Degree of Freedom | *P* Value |
|---|---|---|---|---|---|---|---|---|
| Repeat tumor | FaDu | Control | *in vivo* | *ex vivo* | -3.235 | 0.297 | 2670.483 | 0.000 |
| | | 24 h | *in vivo* | *ex vivo* | 1.988 | 1.287 | 50.761 | 0.129 |
| | SKX | Control | *in vivo* | *ex vivo* | 1.687 | 0.278 | 826.754 | 0.000 |
| | | 24 h | *in vivo* | *ex vivo* | 11.170 | 0.897 | 13.885 | 0.000 |
| | UT-SCC-5 | Control | *in vivo* | *ex vivo* | -0.325 | 0.202 | 1461.697 | 0.107 |
| | | 24 h | *in vivo* | *ex vivo* | -1.457 | 0.397 | 60.007 | 0.001 |
| Repeat ROI | FaDu | Control | *in vivo* | *ex vivo* | -1.465 | 0.285 | 2530.096 | 0.000 |
| | | 24 h | *in vivo* | *ex vivo* | 1.444 | 0.922 | 28.381 | 0.128 |
| | SKX | Control | *in vivo* | *ex vivo* | 1.004 | 0.265 | 1795.228 | 0.000 |
| | | 24 h | *in vivo* | *ex vivo* | 11.291 | 1.037 | 18.506 | 0.000 |
| | UT-SCC-5 | Control | *in vivo* | *ex vivo* | 0.069 | 0.182 | 1451.606 | 0.705 |
| | | 24 h | *in vivo* | *ex vivo* | -1.405 | 0.356 | 19.670 | 0.001 |

**Table S7B**. Summary of the statistical analysis output analyzed by LMEM for $nfoci_g$ for direct comparison between *in vivo* and *ex vivo* datasets. Tumor or ROI was assigned as random effects in the LMEM repeatedly to complete the missing factor for the *in vivo* set-up.

| | Tumor line | | *P* value | | |
|---|---|---|---|---|---|
| | | | SKX | FaDu | UT-SCC-5 |
| Repeat tumor | Fixed effect | Experimental setting | < 0.0001 | 0.206 | 0.022 |
| | Random effect | Inter-tumoral | 0.051 | 0.004 | 0.038 |
| | | Inta-tumoral inter-specimen | 0.033 | < 0.0001 | 0.0056 |
| | | Inta-tumoral intra-specimen | < 0.0001 | < 0.0001 | < 0.0001 |
| Repeat ROI | Fixed effect | Experimental setting | < 0.0001 | 0.159 | 0.016 |
| | Random effect | Inter-tumoral | 0.019 | 0.003 | 0.021 |
| | | Intra-tumoral inter-specimen | 0.000 | < 0.0001 | 0.003 |
| | | Intra-tumoral intra-specimen | 0.009 | < 0.0001 | < 0.0001 |
